# Left Ventricular Diverticulum Mimicking Ventricular Septal Defect During Acute Coronary Syndrome: A Case Report

**DOI:** 10.7759/cureus.24553

**Published:** 2022-04-28

**Authors:** Alejandro J Quiroz Alfaro, Iván J Rodríguez Acosta, María P Gutiérrez Noguera, José G Pinto Quiroz

**Affiliations:** 1 Intensive Care Unit, Clinica Erasmo, Valledupar, COL; 2 Medicina y ciencias de la salud, Universidad Colegio Mayor de Nuestra Señora del Rosario, Bogotá D.C, COL; 3 Cardiology, Instituto Cardiovascular del Cesar, Valledupar, COL

**Keywords:** differential, interventricular communication, ventricular aneurysm, cantrell syndrome, cardiac diverticulum

## Abstract

Left ventricular diverticulum is a rare cardiac congenital anomaly. It is believed to be caused by the impaired development of the endocardial tube during the fourth week of embryologic development. It contains endocardium, myocardium, and pericardium and displays normal contraction. We hereby report a case of a patient with a left ventricular apical diverticulum who presented with unstable angina and was initially misdiagnosed as a ventricular septal defect. Since there were no complications or symptoms associated with the anomaly, the management was conservative. This manuscript also highlights the importance of using multiple diagnostic imaging modalities to exclude differential diagnoses.

## Introduction

Left ventricular diverticulum (LVD) is a rare cardiac congenital anomaly. It is believed to be caused by the impaired development of the endocardial tube during the fourth week of embryologic development and is characterized by a usually asymptomatic outpouching of the cardiac chamber, which contains endocardium, myocardium, and pericardium displaying normal contraction [1,2]. Literature review reports the first description of this anomaly in 1816. Its estimated prevalence is around 0.04% and 0.7% [3,4]. The most common location for the LVD is the apex of the heart in almost 60% of cases and is related to other malformations in up to 70% of affected individuals. A ventricular septal defect is the most common intracardiac anomaly, with midline defects the most common extracardiac malformation. It is also found in association with Cantrell syndrome (abdominal wall defect, sternal-diaphragmatic defect, apical LVD, absence of the inferior pericardial membrane) [1,3]. Some authors subdivide them into two categories: fibrous and muscular types; however, since the fibrous ones lack the myocardial layer, these are considered pseudo-diverticulum by some other authors. Its location can also be used to subclassify them as apical vs non-apical. The muscular type is the most commonly found in the apical location while the fibrous type is usually subvalvular [4]. LVD is mostly encountered in children. Even though rare, LVD can be associated with morbidities and mortality. We present a challenging case of an asymptomatic LVD evidenced by cardiac angiotomography which was initially misdiagnosed as a ventricular septal defect using two-dimensional (2D) echocardiography. We believe that our case raises the awareness of an atypical presentation of such a rare congenital anomaly and emphasizes the importance of cardiac diagnostic imaging in the exclusion of differential diagnoses, including ventricular septal defect.

## Case presentation

**Figure 1:**
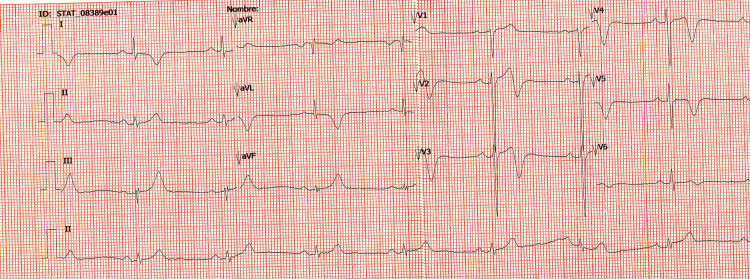
Twelve-lead electrocardiogram showing sinus bradycardia, J point non-significant elevations on V2 and V3 with abnormal progression of R waves in precordial leads

We present here the case of a 60-year-old Hispanic man with a known history of hypertension and dyslipidemia, taking lisinopril 20 mg/day per os (PO), carvedilol 12.5 mg PO q12hr, and atorvastatin 40 mg PO qDay. No relevant allergies or family history was present. The patient presented to the outpatient cardiologist for follow-up. Two weeks prior to the current presentation, the patient presented to our institution’s emergency services and was diagnosed with unstable angina since he had chest pain, negative cardiac enzymes, and inverted T waves on V3 and V4 leads. At that time despite adequate pharmacological management, the chest pain persisted; and therefore, the patient was taken to the angiography suite where coronary angiography was performed, revealing proximal segment stenosis of 98% of the left anterior descending artery (LAD) and an occlusion (100%) of the medial-proximal segment of the LAD. Angioplasty was performed and two drug-eluting stents were placed. Five days later, the patient was discharged. Fourteen days later from the angioplasty, during the outpatient consultation, the patient presented with the sudden onset of oppressive chest pain and dyspnea. The specialist immediately performed an echocardiogram (since no electrocardiograph was available at the doctor's office), evidencing what was considered at that moment a ventricular septal defect, and the patient was transferred to our hospital. In the emergency room, his blood pressure was 145/98 mmHg, heart rate 115 bpm, and a physical examination revealed no rubs or murmurs. In view of his history of hypertension, dyslipidemia, and two recently placed drug-eluting stents in the LAD, we suspected the possibility of acute in-stent thrombosis, as our first differential diagnosis. Another possible differential diagnosis could have entertained a pulmonary embolism. Laboratory results revealed a negative high-sensitivity troponin T assay. Chest X-ray was normal. ECG showed sinus bradycardia, and J point non-significant elevations on V2 and V3 with the abnormal progression of R waves in the precordial leads (Figure 1).

**Figure 2:**
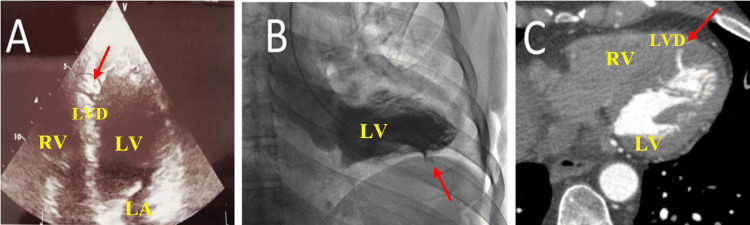
A: Apical four-chamber view showing left ventricular diverticulum. B: Left ventricular ventriculogram showing left ventricular diverticulum. C: Cardiac angiotomography showing left ventricular diverticulum LV=left ventricle; LA=left auricle; RV=right ventricle; LVD=left ventricular diverticulum; Red arrows=pointing LVD

Outpatient 2D transthoracic echocardiography suspected a small 3 mm ventricular septal defect possibly secondary to a complication of a thrombosed stent or an occluded coronary artery; however, this finding was afterward confirmed to be an LVD (Figure 2A).

Coronary angiography exhibited two permeable stents in the anterior descending artery, and no stenotic lesions or occlusions (Videos 1, 2, 3).

**Video 1:** Left coronary angiography Coronary angiography exhibited two permeable stents in the anterior descending artery, and no stenotic lesions or occlusions

 

**Video 2:** Left circumflex artery selective angiography Coronary angiography exhibiting no stenotic lesions

 

**Video 3:** Right coronary artery selective angiography Coronary angiography exhibiting no stenotic lesions

The left ventricular ventriculogram showed a normally contracting left ventricular outpouching that required a cardiac angiotomography for better characterization (Figure 2B, Video 4).

**Video 4:** Left ventricular ventriculogram Left ventricular ventriculogram showing a normally contracting left ventricular outpouching that required a cardiac angiotomography for better characterization

Cardiac angiotomography perfectly delineated the LVD, confirming the diagnosis (Video 5).

**Video 5:** Cardiac angiotomography Cardiac angiotomography perfectly delineating the left ventricular diverticulum

Our final diagnosis was LVD with unstable angina. After initial stabilization with adequate pharmacotherapy, interventional cardiology was consulted to determine the most appropriate subsequent treatment. Since there were no complications associated with the diverticulum, the patient was maintained on conservative treatment and antihypertensive therapy. Four days later, he was discharged on lisinopril, carvedilol, and diuretics at maximum doses, and atorvastatin and dual antiplatelet therapy with clopidogrel and aspirin. At the two-month follow-up, the patient remains asymptomatic with adequate control of his hypertension and dyslipidemia with the previously indicated pharmacotherapy.

## Discussion

LVD differential diagnoses include left ventricular aneurysms, pseudoaneurysms, ventricular clefts, and ventricular septal defect. It is different from a ventricular aneurysm since aneurysmal walls are mostly fibrotic with isolated atrophic myocardial fibers, exhibiting a paradoxical motion. The distinction from the pseudoaneurysm relies on the identification of a contained ruptured ventricular free wall by the pericardium [2,3]. The cleft is localized in the compacted myocardial layer and with contractions usually gets obliterated during systole. These characteristics also make clefts difficult to visualize using echocardiograms [5]. A ventricular septal defect can also be mistaken with LVD (yet it is the most commonly associated defect) because of the difficult visualization of the apex and surrounding structures using an apical four-chamber view. In 2D echocardiograms, the depth of the apical LVD can be mistaken for a complete septal defect as seen in our case. Usually, LVD carries a good prognosis as it is mostly asymptomatic; however, complications like embolic stroke, aortic insufficiency, infective endocarditis, rupture, and arrhythmias can develop [6,7]. Subaortic location, male gender, and a fibrous type are the three major independent risk factors for developing complications [7]. Spontaneous regression, even though rare, has also been reported [6]. Surgical resection of the anomaly and chamber closure is an option, especially if the diverticular neck diameter is ≥2 cm. Another option that has been reported is the closure using a percutaneous approach with a persistent ductus arteriosus closure device. Depending on the feasibility and location, neck diameter, symptoms, complications, and the patient prognosis are also considered when the decision to surgically treat is made [6,7]. In our patient, we believe that the ventricular outpouching was indeed an LVD. The findings that mainly support this diagnosis are the normal contraction of the outpouching during systole (observed on the left ventricular ventriculogram), the absence of acute stent thrombosis or other complications associated with myocardial infarction, and the perfectly delineated morphology of the outpouching on the cardiac angiotomography. Since our patient did not have any complications or symptoms related to the LVD, our approach was conservative management. Our case emphasizes the importance of cardiac diagnostic imaging when diagnosing LVD and excluding other differential diagnoses since a cardiac diverticulum can be overlooked using echocardiography or be mistaken with another kind of ventricular wall defect or outpouching. Like in our case, the acoustic window led to the misinterpretation of the ventricular outpouching mimicking a complete septal defect. We hereby encourage the usage of other diagnostic modalities such as ventriculography or angiotomography as a confirmatory diagnostic method of ventricular wall defects or outpouchings.

## Conclusions

LVD is a rare congenital anomaly and a diagnostic challenge. Since it can be asymptomatic, diagnosing this condition requires a high degree of suspicion since it can be easily overlooked. Therefore, understanding the role of multimodal diagnostic imaging in diagnosing congenital malformations is crucial because as observed in our patient a single diagnostic imaging approach like transthoracic echocardiography has its limitations, such as acoustic window quality, depth, and location of the defect. This can prevent misdiagnosing and inadequate treatment.

LVD treatment is based on individual risk factors, prognosis, and complications. Potential treatments range from closure using a transcatheter device to the surgical resection of the ventricular defect. Males, fibrous type, and subaortic location are the main risk factors associated with complications, and therefore, the most benefited from surgical approaches or any treatment at all. Since there are no established treatment guidelines, the decision for a surgical approach should consider all factors on a case-by-case basis. Conservative management is the usual approach in patients lacking symptoms and risk factors.
